# Whole Exome Sequencing for a Patient with Rubinstein-Taybi Syndrome Reveals *de Novo* Variants besides an Overt *CREBBP* Mutation

**DOI:** 10.3390/ijms16035697

**Published:** 2015-03-11

**Authors:** Hee Jeong Yoo, Kyung Kim, In Hyang Kim, Seong-Hwan Rho, Jong-Eun Park, Ki Young Lee, Soon Ae Kim, Byung Yoon Choi, Namshin Kim

**Affiliations:** 1 Department of Psychiatry, Seoul National University Hospital, Seongnam, Gyeonggi 463-707, Korea; E-Mails: hjyoo@snu.ac.kr (H.J.Y.); iambabyvox@snu.ac.kr (I.H.K.); bulls18@snu.ac.kr (J.-E.P.); 2 Department of Psychiatry, Seoul National University, College of Medicine, Seoul 110-744, Korea; E-Mail: twinwif2@snu.ac.kr; 3 Epigenomics Research Center, Genome Institute, Korea Research Institute of Bioscience and Biotechnology, Daejeon 305-806, Korea; E-Mail: kkyung412@gmail.com; 4 Department of Biomedical Informatics, Ajou University, School of Medicine, Suwon 443-749, Korea; E-Mail: kiylee@ajou.ac.kr; 5 Simulacre Modeling Group, Seoul 140-897, Korea; E-Mail: shrho@simulacre.re.kr; 6 Department of Pharmacology, Eulji University College of Medicine, Daejeon 301-746, Korea; E-Mail: sakim@eulji.ac.kr; 7 Department of Biomedical Science, Ajou University Graduate School of Medicine, Suwon 443-749, Korea; 8 Department of Otolaryngology, Seoul National University Hospital, Seongnam, Gyeonggi 463-707, Korea

**Keywords:** Rubinstein-Taybi syndrome (RSTS), Autism spectrum disorder (ASD), *de novo* variants, Tenascin C (*TNC*) gene, insulin-like growth factor-binding protein, acid labile subunit (*IGFALS*) gene, CREB (cAMP response element binding protein) binding protein (*CREBBP*) gene

## Abstract

Rubinstein-Taybi syndrome (RSTS) is a rare condition with a prevalence of 1 in 125,000–720,000 births and characterized by clinical features that include facial, dental, and limb dysmorphology and growth retardation. Most cases of RSTS occur sporadically and are caused by *de novo* mutations. Cytogenetic or molecular abnormalities are detected in only 55% of RSTS cases. Previous genetic studies have yielded inconsistent results due to the variety of methods used for genetic analysis. The purpose of this study was to use whole exome sequencing (WES) to evaluate the genetic causes of RSTS in a young girl presenting with an Autism phenotype. We used the Autism diagnostic observation schedule (ADOS) and Autism diagnostic interview revised (ADI-R) to confirm her diagnosis of Autism. In addition, various questionnaires were used to evaluate other psychiatric features. We used WES to analyze the DNA sequences of the patient and her parents and to search for *de novo* variants. The patient showed all the typical features of Autism, WES revealed a *de novo* frameshift mutation in *CREBBP* and *de novo* sequence variants in *TNC* and *IGFALS* genes. Mutations in the *CREBBP* gene have been extensively reported in RSTS patients, while potential missense mutations in *TNC* and *IGFALS* genes have not previously been associated with RSTS. The *TNC* and *IGFALS* genes are involved in central nervous system development and growth. It is possible for patients with RSTS to have additional *de novo* variants that could account for previously unexplained phenotypes.

## 1. Introduction

Rubinstein-Taybi syndrome (RSTS, OMIM 180849, 613684), also known as the broad thumb-hallux syndrome, is a rare condition with a prevalence of 1 in 125,000–720,000 births [1,2]. The syndrome is characterized by a group of well-defined clinical features including characteristic facial, dental, and limb dysmorphology, and growth retardation [3]. RSTS can also affect multiple internal organs including the heart, kidney, eyes, ears, and skin [4]. Patients are known to have an increased risk of developing non-cancerous and cancerous tumors, leukemia, and lymphoma [5]. Mental retardation and learning difficulties are other typical findings of RSTS. The average IQ of the patients is 35–50, although cognitive functioning outside this range has also been documented [6].

The diagnosis of RSTS is essentially a clinical diagnosis based on the features described above [7]. Most RSTS cases are sporadic and are caused by *de novo* mutations [8]. Cytogenetic or molecular abnormalities can be detected in only 55% of patients with RSTS [9]. Missense/non-sense mutations in genes encoding the CREB-binding protein (*CREBBP*) or the E1A-binding protein (*EP300*) are the most common, although micro-deletions in chromosome 16p13.3 and other rare mutations have also been reported as genetic causes of this condition [8,10,11,12,13,14,15]. Nevertheless, genetic analyses for identifying mutations in RSTS have often yielded inconsistent results, probably because of inadequacies in the methods used for the analyses, and many mutations remain unconfirmed. Previous studies examining genetic mutations in RSTS have mainly used fluorescence *in situ* hybridization (FISH) [16,17] or comparative genomic hybridization (CGH) array analyses [14]. Whole genome or exome sequencing (WES) methods have rarely been used. The purpose of this study was to use WES to evaluate the genetic causes of RSTS in a young girl presenting with RSTS and Autism spectrum disorder (ASD).

## 2. Results

### 2.1. Physical Characteristics

The typical dysmorphic features of RSTS were observed in the patient’s face and mouth: Microcephaly (head circumference of 32 cm (approximately 3rd to 5th percentile) at birth and 47.8 cm (below the 3rd percentile) at her current age (6.75 years), highly arched eyebrows, long eyelashes, downward-slanting palpebral fissures, a broad nasal bridge, a beaked nose with the nasal septum extending well below the alae, a pouting lower lip, mild micrognathia, and dental crowding.

The patient had big toes that were short and broad, and mild clinodactyly of the fifth digits of both hands. The second, third, and fourth digits of the right hand had short distal phalanges, and the third and fourth digits of the left hand had broad distal phalanges. She also had a curly toe on the fourth digit of both feet and hallux valgus in the right foot. X-ray radiographs showed bilateral pes varus deformities and bilateral big-toe soft tissue thickening. There were abnormal accessory physes on the side of the distal phalanx, as well as other abnormalities in the physes of the big toes of both feet. Hand X-ray radiographs showed delayed carpal bone ossification in both hands and prominent thickening of the soft tissue of all fingers.

**Figure 1:**
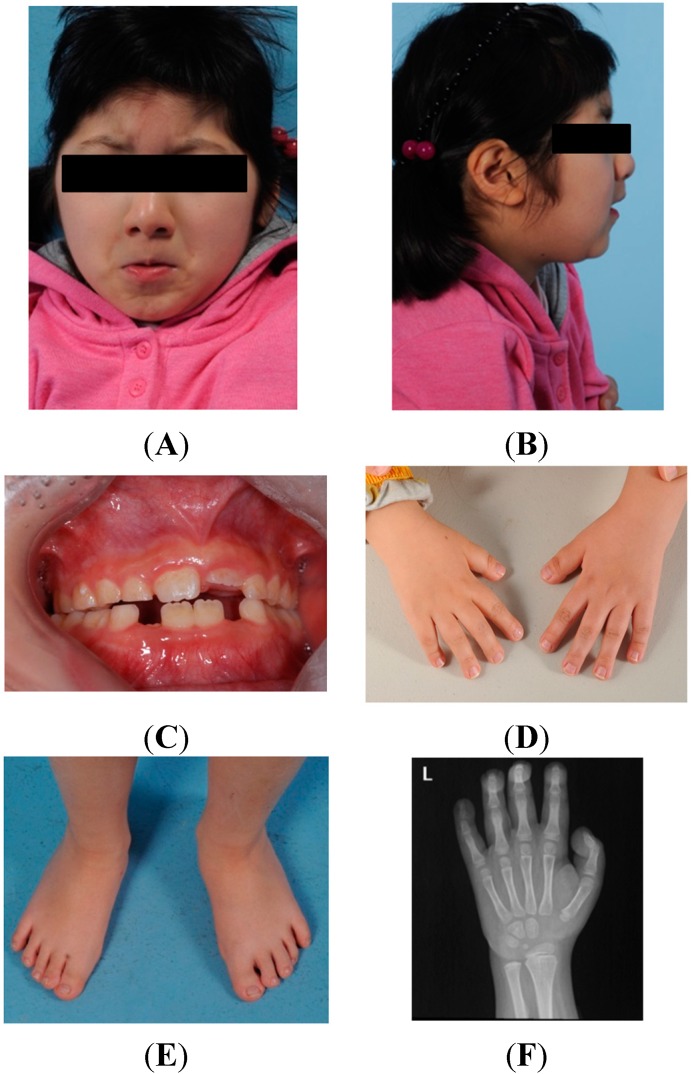

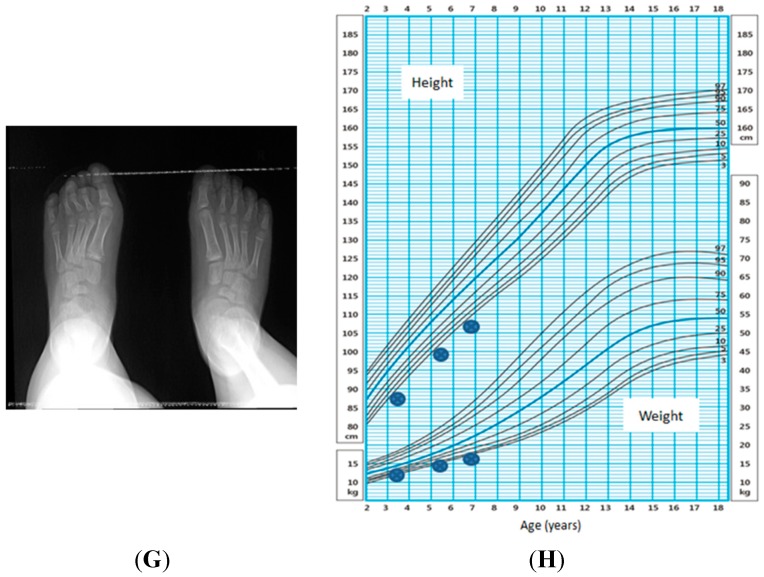
Physical characteristics of the proband. Patient’s eyes are covered for reasons of confidentiality. Typical facial dysmorphic features can be observed in (**A**,**B**), including highly arched eyebrows, a broad nasal bridge, beaked nose with the nasal septum extending well below the alae, a pouting lower lip, and mild micrognathia. Dental crowding is shown in (**C**); The hand and feet abnormalities are shown in (**D**,**E**), showing short and broad big toes and thumbs, as well as mild clinodactyly on the 5th digit of both hands. The 2nd, 3rd, 4th digits of the right hand had short distal phalanges and the 3rd, 4th digits of the left hand had broad distal phalanges. The X-ray of the left hand shows delayed carpal bone ossification and prominent thickening of the soft tissue of all fingers (**F**); The X-ray of the feet shows bilateral pes varus deformities and abnormalities in the physes of big toe metatarsal bones (**G**); Growth curve (**H**) shows marked growth retardation in the proband (blue dot) superimposed on the normal growth curve of Korean girls of age 2 to 18 (black line). The growth curve shows the height and weight changes of the patient over time. These values never exceeded the third percentile since birth. Data for normal growth curve came from the Korea Center for Disease Control and Prevention.

Marked growth retardation and poor weight gain were prominent in this patient from birth until her latest evaluation. Her height was 106.3 cm (below the 3rd percentile) and her weight was 17.2 kg (3rd percentile). A videofluoroscopic swallowing study revealed difficulty in swallowing. Furthermore, serum prealbumin and zinc levels were low. Other physical findings in the patient included anomalies of the eye (congenital cataract and nasolacrimal duct obstruction) and skin (pilomatrixoma on the left periauricular area, hypertrichosis on the forehead, anterior chest, and back, as well as nevus depigmentosus on the right calf). Gross hematuria had previously been found at five years of age, although a kidney ultrasound examination showed no abnormal findings. Results of cardiac evaluation were within normal limits. The characteristic physical features and growth curve of the patient are shown in Figure 1.

### 2.2. Neuropsychiatric and Behavioral Features

The patient showed pronounced developmental delay with no meaningful vocabulary. She also showed moderate mental retardation with a full-scale intelligence quotient (IQ) score of 37 on the Korean Wechsler Preschool and Primary Scale for Intelligence (K-WPPSI) and a score of 45 on the Leiter International Performance Scale. Her adaptive functioning level was equivalent to a child of approximately 3–5 years of age. She had shown the typical behavioral characteristics of Autism from early infancy, including abnormalities in communication and social interaction, along with repetitive behavior and restricted interests. Her scores surpassed the diagnostic thresholds on all categories of both the ADOS and ADI-R. She presented the distinctive mannerism of repetitively waving her hand whenever emotionally disturbed. Scores for other questionnaires also supported the diagnosis of Autism including a score of 15 on the social communication questionnaire (SCQ) and a score of 113 on the social component of the social responsiveness scale (SRS). Further behavioral characteristics included marked hyperactivity, short attention span, withdrawal, and poor motor coordination. Frequent snoring was reported, although the possibility of obstructive sleep apnea was deemed low. Brain magnetic resonance imaging (MRI) showed mild pachygyria. Electroencephalogram (EEG) showed intermittent diffuse high-amplitude semirhythmic 1–1.5-Hz delta activity and one episode of diffuse 1.5-Hz rhythmic, synchronous bifrontal δ/ζ activity lasting up to 20 s.

### 2.3. Genetic Variants

Cytogenetic analyses showed normal karyotypes in the proband and both parents. Using in-depth statistics, we obtained a mean read-depth coverage of 120× over the targeted exome. Each exome contained more than 120,000 total variant calls. An average of 111 million individual paired-end reads of 100 bp were aligned to the human reference genome. More than 94% of targeted exon regions were covered with at least 10× depth, which is sufficient for variant discovery.

We found three *de novo* variants after filtering for all common variants in the dbSNP (except clinically associated variants) and an in-house control database comprising normal healthy individuals. We performed a visual inspection of read alignments using the Integrative Genomics Viewer (IGV) browser, and then removed “Benign” and “Likely Benign” variants after classification. A summary of the detected variants is described in Table S1.

**Figure 2:**
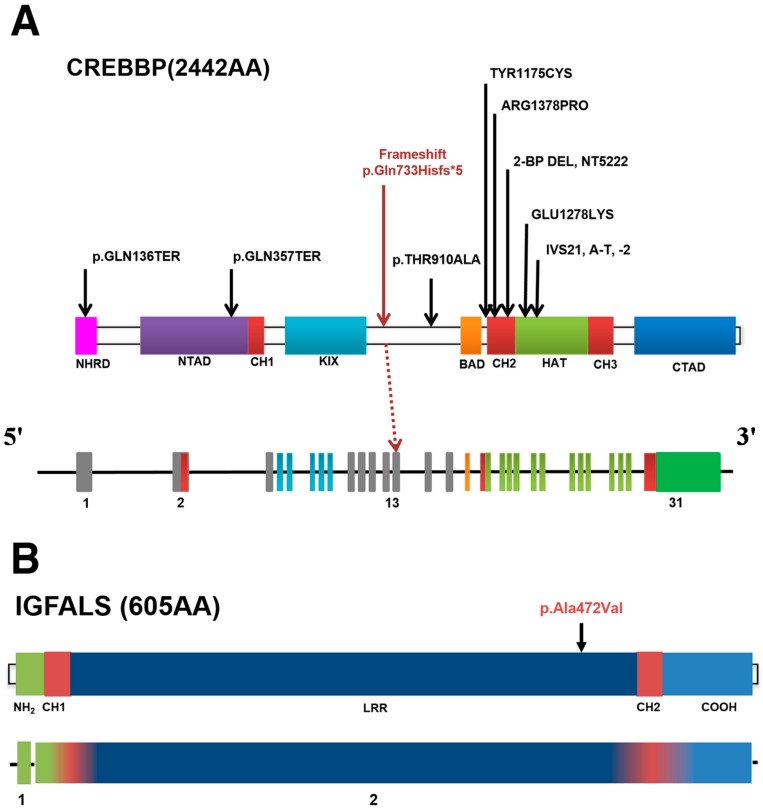

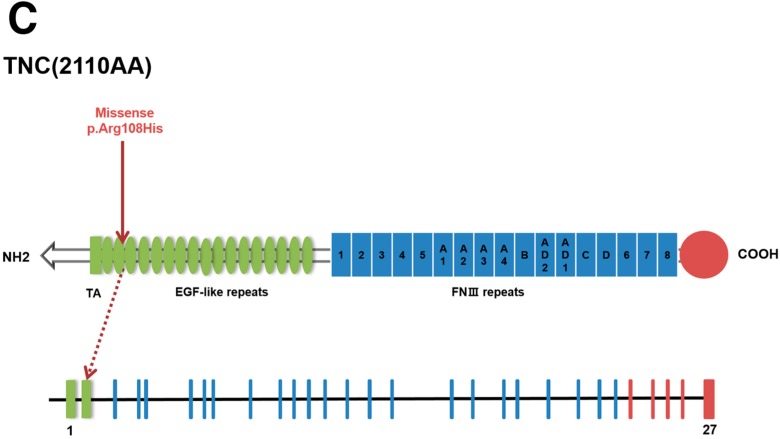
Schematic of *de novo* variants of *CREBBP*, *IGFALS*, and *TNC* in RSTS. (**A**) Genomic alterations and schematic drawing of proteins showing the predicted effects of selected missense mutations identified in *CREBBP*. The p.Thy1174Cys and p.Thr910Ala substitutions predicted by missense mutations do not affect known functional domains and are associated with a mild phenotype compared with the classic phenotype expected for mutations at other sites. The frameshift mutation p.Gln733Hisfs*5 is predicted to interrupt all of the following functional domains: BAD, CH2, HAT, CH3, and CTAD; (**B**) The *IGFALS* mutation site affects the LRR domain and (**C**) the *TNC* mutation one of the EGF-like repeats. The *de novo* variants are shown in red. CREBBP: CREB (cAMP response element-binding protein) binding protein; IGFALS: Insulin-like growth factor)-binding protein, acid labile subunit; TNC: Tenascin C; Protein domain names: (**A**) NHRD: Nuclear receptor-binding and receptor-interacting domain; NTAD: Amino-terminal transactivation domain; CH1: Cys/His-rich region; CREB/KIX: CREB-binding domain; BROMO/BRD: Bromo domain; HAT: Histone acetyltransferase domain; CH2: Cys/His-rich region 2; CH3: Cys/His-rich region 3; CTAD: *C*-terminal transactivation domain; (**B**) NH2: *N*-terminal; CH1: Cys-rich region 1; LRR: Leucine rich repeats; CH2: Cys-rich region 2; COOH: *C*-terminal; (**C**) TA: *N*-terminal tenascin assembly domain; EGF-like repeats: Epidermal growth factor-like repeats; FNIII repeats: Fibronectin type III-like repeats; FN Globe: *C*-terminal fibrinogen globe

**Figure 3:**
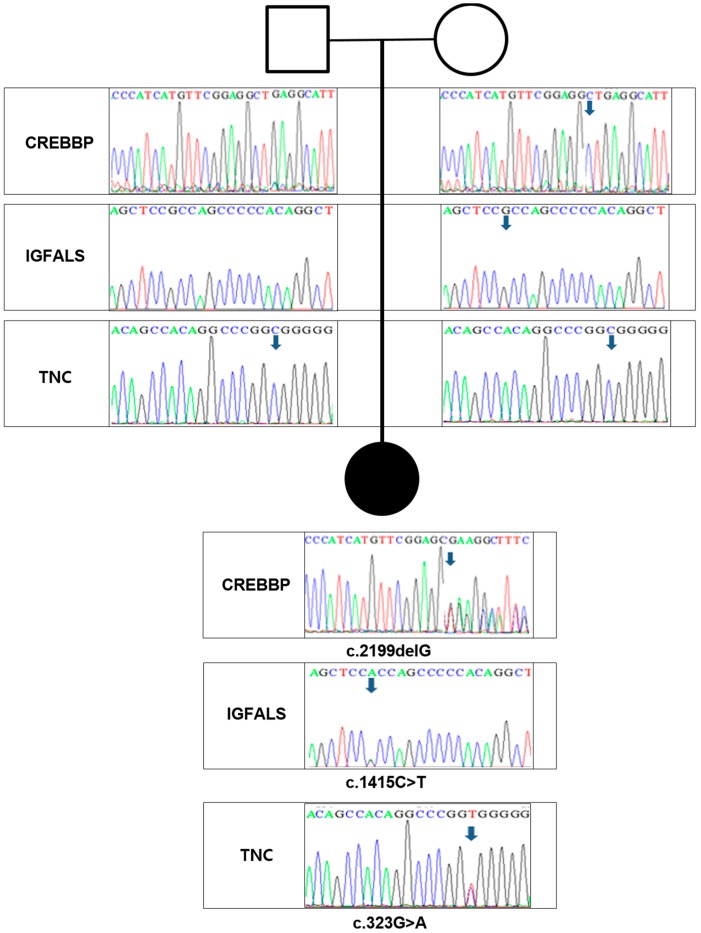
Sequences of *CREBBP*, *IGFALS*, and *TNC* for the family trio. A downward arrow indicates the mutated residue and nucleotides are shown in colored, single-letter codes. The black circle indicates the proband.

Three *de novo* variants were validated by Sanger sequencing: a frameshift mutation in *CREBBP* (c.2199delG), and missense mutations in *TNC* (c.323G>A) and *IGFALS* (c.1415C>T). The results of family sequencing analyses are shown in Figure 2 and the properties of the *de novo* variants are summarized in Table 1 and Figure 2. The genetic variants of the proband and both parents are shown in Figure 3.

**Table 1:** The three *de novo* variants validated by Sanger Sequencing.

Gene	*TNC*	*IGFALS*	*CREBBP*
**Chromosome**	chr9	chr16	chr16
**Position**	117852975	1841118	3823901
**Reference Allele**	C	G	C
**Alternative Allele**	T	A	–
**Mutation Type**	Missense	Missense	Frameshift
**Sequence Variant**	c.323G>A	c.1415C>T	c.2199delG
**Protein Variant**	p.Arg108His	p.Ala472Val	p.Gln733Hisfs*5
**phyloP Score**	2.369	0.598	1.492
**GERP Score**	1200.8	2536.4	476.2
**SIFT Score**	0	0.30	–
**Mutation Assessor**	1.87	0.995	–
**Classification ^1^**	Likely pathogenic ^2^	Variants of unknown significance	Pathogenic

^1^ Variant Classification is based on the recommendations of Ambry Genetics, but pathogenicity of *de novo* variants in *TNC* and *IGFALS* are not certain; ^2^ *TNC* c.323G>A is reported in dbSNP as rs151119387, but MAF/MinorAlleleCount = 0.0002/1. Overall MAF in 1000 genomes, ESP6500, ExAc database is less than 0.0003. *TNC*: Tenascin C; *IGFALS*: Insulin-like growth factor-binding protein, acid labile subunit; *CREBBP*: CREB (cAMP response element-binding protein) binding protein; phyloP: Phylogenetic *p*-values; GERP: Genomic evolutionary rate profiling; SIFT: Sorting intolerant from tolerant.

**Figure 4:**
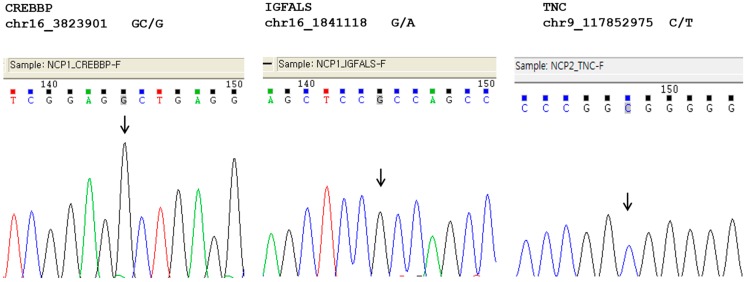
Validation results for *de novo* variants in *CREBBP*, *IGFALS*, and *TNC* in 80 normal Korean subjects. The downward arrow indicates the residue of *de novo* variants.

None of the observed sequence changes were observed in normal Korean subjects. The validation results from the normal population are shown in Figure 4.

## 3. Discussion

We have shown that *de novo* variants in the *CREBBP*, *TNC*, and *IGFALS* genes are potential causes for RSTS by analyzing the whole genome of a female patient with RSTS. In WES analyses, we observed *de novo* variants in the *CREBBP*, *TNC*, and *IGFALS* genes. The girl showed typical morphological findings of RSTS, including abnormal features of the face, fingers, and toes, as well as growth retardation. Major characteristics of this patient were severe developmental/behavioral problems together with ASD with typical features that fulfilled the diagnostic criteria in multiple domains including communication, social interaction, specialized interests, and repetitive behavior.

Developmental/behavioral problems are common in patients with RSTS, with the most typical symptoms being moderate to severe mental retardation and language impairment [8,18]. There are no definitive reports regarding the specific frequency of psychiatric disorders in RSTS due to its low prevalence. In terms of autistic behavior, only a few reports have described this specific psychopathology associated with RSTS. In one relatively large-scale study exploring the socio-behavioral characteristics of RSTS, this syndrome was associated with behavioral problems such as a short attention span, motor stereotypies, and poor coordination [19]. These can also be part of the behavioral phenotype of ASD. The studies that have reported Autism-like phenotypes in patients with RSTS describe milder and atypical forms of ASD (*i.e*., pervasive developmental disorder not otherwise specified; PDD NOS) in only a small proportion (8%) of the subjects [20,21,22,23].

Mutations in the *CREBBP* gene are one of the most commonly reported genetic etiologies of RSTS (Table S2). *CREBBP* mutations are found in 50%–60% of RSTS cases, whereas *EP300* (OMIM 602700) mutations are found in 5% of the cases [7,8]. RSTS is assumed to be a genetically heterogeneous disorder which is caused by mutations in the *CREBBP* gene as well as in other genes in up to 50% of cases [2]. The *CREBBP* mutations are heterogeneous, as 160 different mutations have been identified (Leiden Open Variation Database v.2.0 Build 35) [24]. The *CREBBP* gene is ubiquitously expressed and is involved in the transcriptional co-activation of many transcription factors [25]. *CREBBP* is also involved in multiple signaling pathways and in other cellular functions such as DNA repair, cell growth, differentiation, apoptosis, and tumor suppression [26]. The CREBBP protein plays an important role in regulating cell growth and division and is essential for normal fetal development. If one copy of *CREBBP* is deleted or mutated, cells make only half the normal amount of CREBBP protein. This reduction in the amount of this protein is thought to disrupt normal development before and after birth. The *CREBBP* mutation is known to be associated with more severe symptomatology than the *EP300* mutations [24]. Based on previous studies, the severe cognitive symptomatology of the subject of this study, including the ASD phenotype, could be attributed to the *CREBBP* mutation.

We can infer that the *de novo* sequence changes of the *TNC* and *IGFALS* genes, featuring as missense mutations observed in our study could potentially explain any additional phenotypes in patients with RSTS, but experimental validation is required to establish disease causation. Tenascin C (TNC), encoded by *TNC*, is a glycoprotein expressed in the extracellular matrix of various tissues during development, disease, or injury, as well as in restricted areas of the central nervous system involved in neurogenesis [27]. TNC expression changes during development until adulthood. In the developing central nervous system, it is involved in regulating the proliferation of both oligodendrocyte precursor cells and astrocytes [28]. TNC expression by radial glia precedes the onset of gliogenesis, and is thought to drive astrocyte differentiation. In the adult brain, TNC expression is downregulated in all areas except the hypothalamus and areas that maintain neurogenesis into adulthood [28]. Male-specific association signals have been found in the intronic region of the *TNC* gene [29]. A missense mutation c.5317G>A (p.V1773M) in exon 19 of the *TNC* gene was recently reported as a causative gene for nonsyndromic hearing loss in family-based exome sequencing and linkage analyses [30]. Based on the fact that pronounced-to-profound bilateral hearing loss or deafness is diagnosed more commonly in ASD than in the general population, the causality of deafness and ASD might have a partial overlap [31]. This study suggests that mutations in the *TNC* gene could contribute to sensory impairment in ASD, including hearing loss. The patient with RSTS in this study showed slight hearing problems (data now provided), but it is still not clear which specific aspect of the patient’s phenotype can be attributed to the missense mutations in *TNC.*

To date, the presence of a genetic mutation in *CREBBP* has been linked with the behavioral phenotype in RSTS. For example, poor coordination is more prevalent in patients with RSTS and in subjects with genetic abnormalities in *CREBBP* than in those who have no abnormalities in this gene [19]. Severe cognitive impairments and autistic features have been reported in patients with large deletions in *CREBBP* [32]. This can be explained by the involvement of CREB-dependent gene transcription in long-term memory formation through signaling pathways that include multiple important kinases (protein kinase A, mitogen-activated protein kinase, and calcium/calmodulin-dependent kinase IV (CaMKIV)) in the nervous systems of many species [2]. The mental retardation and cognitive phenotype in patients with RSTS may be derived from impairments to the function of CREB-binding protein [2,13]. The role of the *CREBBP* mutation in long-term memory formation is supported by experiments showing that memory impairments in a *CREBBP*^+/–^ mutant mouse model were ameliorated by phosphodiesterase 4 inhibitors [33]. Our observations suggest that *de novo* variants can have an additive effect and produce more severe and defined neurological/cognitive aberrations in subjects with RSTS.

The acid-labile subunit of insulin-like growth factor-binding protein (IGFALS) encoded by the *IGFALS* gene, is a serum protein that binds to IGFs. The IGFALS protein interacts with growth hormones to increase their half-life as well as their vascular localization [34]. *IGFALS*-dependent growth changes related to sex-specific body characteristics and bone size have been reported previously [35,36]. Defects in this gene cause acid-labile subunit deficiency which manifests as delayed and slow development during puberty [37]. The growth retardation in our patient, which has been present from birth until her current age, might be enhanced by the variants in the *IGFALS* gene.

One limitation of our study is that the analysis was restricted to only a single case. Validation of our results was also performed using relatively small samples from the general population. The precise function of the mutations and variants needs to be validated in future studies.

In conclusion, using WES, we observed *de novo* variants in the *CREBBP*, *TNC*, and *IGFALS* genes in a female patient with RSTS. To our knowledge, this is the first report suggesting that variants in the *TNC* and *IGFALS* genes can produce additional phenotypic effects. We assume that *de novo* variants in the *TNC* gene are related to the neurological phenotype and those in the *IGFALS* gene are related to growth retardation.

## 4. Methods

### 4.1. Ethics Statement

This study was approved by the Institutional Review Board (IRB) of the Seoul National University Bundang Hospital (Seongnam, Gyeonggi, Korea). Written informed consent was obtained from both parents of the patient.

### 4.2. Subject

The patient, a girl aged 6 years and 8 months, was examined in the child psychiatry clinic of the Seoul National University Bundang Hospital. She was thoroughly evaluated to assess problems related to delayed development and dysmorphology. A genetic assessment to diagnose RSTS, a possibility indicated during a previous diagnosis process, was also performed. The patient was the only child of a healthy non-consanguineous couple. Both biological parents were healthy with no clinically significant medical, developmental, or neuropsychological illnesses.

### 4.3. Clinical Evaluation

Thorough physical and laboratory evaluations were performed to confirm the diagnosis of RSTS and obtain detailed clinical and behavioral features. Physical, dental, ophthalmologic, and dermatologic examinations were performed. Chest and limb/digit X-ray radiography, urine and blood tests, a videofluoroscopic swallowing study, kidney ultrasonography, echocardiography, and electrocardiography were all performed as part of the laboratory analyses. Intelligence and adaptive functioning were assessed using the Korean version of the Wechsler preschool and primary scale of intelligence (K-WPPSI), the Leiter international performance scale [6,38], and the Vineland Adaptive Behavior scale (VABS) [39,40]. Two board-certified child psychiatrists evaluated the autistic symptoms. The Autism diagnostic observation schedule (ADOS), Module I [41,42], and the Autism diagnostic interview-revised (ADI-R) [43,44] were used, as well as the social communication questionnaire (SCQ) and social responsiveness scale (SRS) [45,46]. We measured the patients sleep quality using the Pittsburgh sleep quality index (PSQI) [47] and the “STOP” questionnaire (snoring, tiredness during daytime, observed apnea, high blood pressure) [48]. Brain magnetic resonance imaging (MRI) and electroencephalography (EEG) were performed to elucidate potential structural and functional abnormalities in the brain.

### 4.4. Genetic Analyses Using Bioinformatics Tools

Blood samples were drawn from the patient and both biological parents. Genomic DNA was extracted using the DNeasy Blood & Tissue kit (QIAGEN, Valencia, CA, USA). Raw reads in FASTQ format from exome sequencing were aligned to the hg19 reference genome from the UCSC genome browser using the Burrows–Wheeler Aligner (BWA) [49] with default parameters. Aligned reads were processed and polymerase chain reaction (PCR) duplicates were removed with SAMtools [50]. Single-nucleotide variants (SNVs) and insertions/deletions (indels) were identified using the Genome Analysis Toolkit (GATK) [51]. Regions near short indels were realigned using the IndelRealigner function in GATK according to default parameters. SNVs and indels affecting coding sequences or splicing sites were annotated by an in-house custom-made annotation system. All genomic changes were filtered against the Single Nucleotide Polymorphism Database (dbSNP; build 141) and the in-house control database comprising 54 Korean individuals.

We classified the *de novo* variants using the recommendations of Ambry Genetics (Scheme for AD and XD Mendelian disorders) into five categories: Benign, VLB (Variant, Likely benign), VUS (Variant, Unknown significance), VLP (Variant, Likely pathogenic), and Pathogenic mutation. Variants classified as VUS, VLP, and Pathogenic mutation were also confirmed using an IGV browser. To evaluate the mutations in the general population, we also applied Sanger sequencing for these mutations to 80 normal subjects belonging to the Korean ethnic group.

## 5. Conclusions

RSTS is a rare disorder characterized by facial, dental, and limb dysmorphology, growth retardation, and developmental disorders including intellectual disability and Autism spectrum disorder. Although many *de novo* mutations have been proposed as the genetic cause of RSTS, genetic analyses to identify mutations have often yielded inconsistent results due to inadequacies in the analysis methods, and many mutations are yet to be confirmed. The objective of this study was to investigate the genetic background of a young girl with RSTS presenting with an Autism phenotype, using whole exome sequencing (WES). We observed *de novo* variants in the *CREBBP*, *TNC*, and *IGFALS* genes. As far as the authors know, this is the first report to suggest that the *TNC* and *IGFALS* genes might contribute to clinical signs that are uncommon in RSTS patients.

## Supplementary Materials

Supplementary materials can be found at http://www.mdpi.com/1422-0067/16/03/5697/s1.
