# Supplementary material for: Whole Exome Sequencing for a Patient with Rubinstein-Taybi Syndrome Reveals de Novo Variants besides an Overt CREBBP Mutation
Source: Int J Mol Sci. 2015 Mar 11;16(3):5697–713. doi: 10.3390/ijms16035697 (PMC4394500; doi:10.3390/ijms16035697)
Supplement: Supplementary file 1 [file ijms-16-05697-s001.pdf]

Supplementary Information

Table S1. Exome Sequencing results and variant statistics.

Sample	Father	Mother	Daughter
Total reads	118,107,056	105,819,946	111,140,886
Mapped reads	117,000,234	104,605,168	110,159,887
Mapped reads (%)	99	99	99.1
Non-duplicate reads	105,488,263	92,767,292	99,213,228
Non-duplicate reads (%)	89	88	89
On-target reads	95,777,991	81,868,105	88,767,150
On-target reads (%)	81	77	80
Mean depth	141	120	131.58
Bases_above_1 (%)	99	99	99
Bases_above_5 (%)	97	99	97
Bases_above_10 (%)	95	94	95
Bases_above_20 (%)	91	90	90
Total	122,157	120,673	123,392
Homozygous	57,587	57,246	56,173
Heterozygous	63,661	62,644	66,718
Transition/Transversion Ratio (%)	2.25	2.26	2.28
CDS	20,154	19,813	20,318
Utr5	3299	3181	3252
Utr3	3423	3353	3445
Intron	76,807	75,090	77,129
Non_coding_exon	6260	6094	6284
Non_coding_intron	19,412	19,197	19,490
Silent	9934	9694	10,017
Missense	8875	8770	8939
Inframe	226	203	214
Frameshift	119	126	121
Non-sense	70	78	81
Read through	18	17	20
Start codon	27	29	32
Splicing	176	163	172
Junction	59	65	65
NS/SS/Indel	9469	9346	9536

Table S2. Previous reports of *CREBBP* mutations in Rubinstein-Taybi Syndrome.

Index	Phenotype	cDNA Change	A.A^{-1} Variant	Origin	OMIM ID	dbSNP	Reference
1		c.3833A>C	p.Glu1278Ala	*de novo*	-	-	Gervasini, personal communication, Netherlands: Leiden [2]
2		c.4014G>C	p.Leu1338Phe	*de novo*	-	-	Gervasini, personal communication, Netherlands: Leiden [2]
3		c.4216G>T	p.Asp1406Tyr	*de novo*	-	-	Gervasini, personal communication, Netherlands: Leiden [2]
4		c.4281-7C>G	p.? [3]	*de novo*	-	-	Gervasini, personal communication, Netherlands: Leiden [2]
5		c.4444T>G	p.Tyr1482Asp	*de novo*	-	-	Gervasini, personal communication, Netherlands: Leiden [2]
6		c.5060C>T	p.Ser1687Phe	*de novo*	-	-	Gervasini, personal communication, Netherlands: Leiden [2]
7		c.85+1G>T	p.? [3]	*de novo*	-	-	Gervasini, personal communication, Netherlands: Leiden [2]
8		c.223C>T	p.Arg75X	*de novo*	-	-	Gervasini, personal communication, Netherlands: Leiden [2]
9		c.472C>T	p.Gln158X	*de novo*	-	-	Gervasini, personal communication, Netherlands: Leiden [2]
10		c.810_811delTG	p.Gly271fs	*de novo*	-	-	Gervasini, personal communication, Netherlands: Leiden [2]
11		c.1483C>T	p.Gln495X	*de novo*	-	-	Gervasini, personal communication, Netherlands: Leiden [2]
12		c.1515_1521delTGGCCAG	p.Gly506fs	*de novo*	-	-	Gervasini, personal communication, Netherlands: Leiden [2]
13		c.4650_4654delAGAGA	p.Glu1551fs	*de novo*	-	-	Gervasini, personal communication, Netherlands: Leiden [2]
14		c.5790delC	p.Pro1929fs	*de novo*	-	-	Gervasini, personal communication, Netherlands: Leiden [2]
15		c.2461C>T	p.Gln821X	*de novo*	-	-	Gervasini, personal comunication, Netherlands: Leiden [2]
16		c.3858_3859delTG	p.Cys1286fs	*de novo*	-	-	Gervasini, personal comunication, Netherlands: Leiden [2]
17		c.4627G>A	p.Asp1543Asn	*de novo*	-	-	Gervasini, personal comunication, Netherlands: Leiden [2]
18		c.4650_4654delAGAGA	p.Glu1551fs	*de novo*	-	-	Gervasini, personal comunication, Netherlands: Leiden [2]
19		c.3779+3A>T	p.? [3]	*de novo*	-	-	M.J. van Belzen, Netherlands: Leiden [2]
20		c.4394+4A>C	p.? [3]	*de novo*	-	-	M.J. van Belzen, Netherlands: Leiden [2]
21		c.4708G>T	p.Ala1570Ser	*de novo*	-	-	M.J. van Belzen,Netherlands: Leiden [2]
22		c.4885_4887delAAG	p.Lys1629del	*de novo*	-	-	M.J. van Belzen, Netherlands: Leiden [2]
23		c.(?_-204)_798+?del	p.? [3]	*de novo*	-	-	M.J. van Belzen, Netherlands: Leiden [2]
24		c.(?_-204)_798+?del	p.? [3]	*de novo*	-	-	M.J. van Belzen, Netherlands: Leiden [2]
25		c.37A>G	p.Lys13Glu	*de novo*	-	-	M.J. van Belzen, Netherlands: Leiden [2]
26		c.1522C>T	p.Gln508X	*de novo*	-	-	M.J. van Belzen, Netherlands: Leiden [2]
27		c.3837-?_3982+?del	p.? [3]	*de novo*	-	-	M.J. van Belzen, Netherlands: Leiden [2]

Table S2. *Cont.*

Index	Phenotype	cDNA Change	A.A. Variant	Origin	OMIM ID	dbSNP	Reference
28		c.4256_4257delCT	p.Ser1419X	*de novo*	-	-	M.J. van Belzen, Netherlands:Leiden [2]
29	Typical	c.406C>T	p.Gln136X	-	OMIM 0001	rs121434624	Petrij 1995 [13]
30	Typical	c.1066C>T	p.Gln356X	-	OMIM 0002	rs121434625	Petrij 1995 [13]
31	Typical	c.4319_4320delTC	p.Phe1440fs	-	OMIM 0003	rs121434626	Murata 2001 [52]
32	Typical	c.4898_4908del	p.Phe1633fs	-	OMIM 0004	-	Murata 2001 [52]
33	Mild	c.3524A>G	p.Tyr1175Cys	-	OMIM 0005	rs28937315	Bartsch 2002 [53]
34	Typical	c.3832G>A	p.Glu1278Lys	-	OMIM 0006	-	Kalkhoven 2003 [54]
35	Typical	c.3837-2A>T	p.? [3]	-	OMIM 0007	-	Kalkhoven 2003 [54]
36	Mild	c.2728A>G	p.Thr910Ala	-	OMIM 0008	-	Bartsch 2002 [53]
37		c.86_233del	p.Asp29fs	-	-	-	Bartsch 2002 [53]
38		c.1108C>T	p.Arg370X	-	-	-	Bartsch 2002 [53]
39		c.1216+1G>A	p.? [3]	-	-	-	Bartsch 2002 [53]
40		c.1676+1G>A	p.? [3]	-	-	-	Bartsch 2002 [53]
41		c.4304A>T	p.Asp1435Val	-	-	-	Bartsch 2005 [9]
42		c.4991G>A	p.Arg1664His	-	-	-	Bartsch 2005 [9]
43		c.474_493del	p.Val159fs	-	-	-	Bartsch 2005 [9]
44		c.1270C>T	p.Arg424X	-	-	-	Bartsch 2005 [9]
45		c.1891_1895delGCCTA	p.Ala631fs	-	-	-	Bartsch 2005 [9]
46		c.2986G>T	p.Glu996X	-	-	-	Bartsch 2005 [9]
47		c.3639C>A	p.Cys1213X	-	-	-	Bartsch 2005 [9]
48		c.3767_3769delCAC	p.Ser1256X	-	-	-	Bartsch 2005 [9]
49		c.4321dupC	p.Arg1441fs	-	-	-	Bartsch 2005 [9]
50		c.5635C>T	p.Gln1879X	-	-	-	Bartsch 2005 [9]
51		c.6019C>T	p.Gln2007X	-	-	-	Bartsch 2005 [9]
52		c.6044_6050dupGCATGCC	p.Pro2018fs	-	-	-	Bartsch 2005 [9]
53		c.40A>G	p.Arg14Gly	-	-	-	Bentivegna 2006 [10]
54		c.4445A>G	p.Tyr1482Cys	-	-	-	Bentivegna 2006 [10]

Table S2. *Cont.*

Index	Phenotype	cDNA Change	A.A. Variant	Origin	OMIM ID	dbSNP	Reference
55		c.4627G>T	p.Asp1543Tyr	-	-	-	Bentivegna 2006 [10]
56		c.1108C>T	p.Arg370X	-	-	-	Bentivegna 2006 [10]
57		c.1270C>T	p.Arg424X	-	-	-	Bentivegna 2006 [10]
58		c.1984C>T	p.Gln662X	-	-	-	Bentivegna 2006 [10]
59		c.3351_3352dupCC	p.Gln1118fs	-	-	-	Bentivegna 2006 [10]
60		c.3517C>T	p.Arg1173X	-	-	-	Bentivegna 2006 [10]
61		c.3715_3716delAA	p.Lys1239fs	-	-	-	Bentivegna 2006 [10]
62		c.4435G>T	p.Gly1479X	-	-	-	Bentivegna 2006 [10]
63		c.4728+1G>A	p.? [3]	-	-	-	Bentivegna 2006 [10]
64		c.4963delC	p.Leu1655fs	-	-	-	Bentivegna 2006 [10]
65		c.6043delA	p.Ser2015fs	-	-	-	Bentivegna 2006 [10]
66		c.6065_6071delAGCAGGC	p.Gln2022fs	-	-	-	Bentivegna 2006 [10]
67		c.6661A>C	p.Met2221Leu	-	-	-	Coupry 2002 [55]
68		c.2941G>A	p.Ala981Thr	-	-	-	Coupry 2002 [55]
69		c.3698+3A>T	p.? [3]	-	-	-	Coupry 2002 [55]
70		c.4561-5C>G	p.? [3]	-	-	-	Coupry 2002 [55]
71		c.6728C>T	p.Ala2243Val	-	-	-	Coupry 2002 [55]
72		c.4559A>G	p.Lys1520Arg	-	-	-	Coupry 2002 [55]
73		c.68C>A	p.Ser23X	-	-	-	Coupry 2002 [55]
74		c.139delAinsTCATCATGAGCTG	p.Asn47fs	-	-	-	Coupry 2002 [55]
75		c.840dupT	p.Ser281X	-	-	-	Coupry 2002 [55]
76		c.1108C>T	p.Arg370X	-	-	-	Coupry 2002 [55]
77		c.1237C>T	p.Arg413X	-	-	-	Coupry 2002 [55]
78		c.2045dupA	p.Pro683fs	-	-	-	Coupry 2002 [55]
79		c.2827delC	p.Gln943fs	-	-	-	Coupry 2002 [55]
80		c.3096dupT	p.Lys1033X	-	-	-	Coupry 2002 [55]

Table S2. *Cont.*

Index	Phenotype	cDNA Change	A.A. Variant	Origin	OMIM ID	dbSNP	Reference
81		c.3369_3369+6delinsCA	p.? [3]	-	-	-	Coupry 2002 [55]
82		c.3805A>T	p.Lys1269X	-	-	-	Coupry 2002 [55]
83		c.4280+2T>C	p.? [3]	-	-	-	Coupry 2002 [55]
84		c.4398T>A	p.Tyr1466X	-	-	-	Coupry 2002 [55]
85		c.4492C>T	p.Arg1498X	-	-	-	Coupry 2002 [55]
86		c.4945delA	p.Ile1649fs	-	-	-	Coupry 2002 [55]
87		c.6127C>T	p.Gln2043X	-	-	-	Coupry 2002 [55]
88		c.1011dupA	p.Gln338fs	-	-	-	D.J.M. Peters, personal communication, Netherlands: Leiden [2]
89		c.1114C>T	p.Gln372X	-	-	-	D.J.M. Peters, personal communication, Netherlands: Leiden [2]
90		c.1412_1415delGTAA	p.Ser471fs	-	-	-	D.J.M. Peters, personal communication, Netherlands: Leiden [2]
91		c.2254C>T	p.Gln752X	-	-	-	D.J.M. Peters, personal communication, Netherlands: Leiden [2]
92		c.2678C>A	p.Ser893X	-	-	-	D.J.M. Peters, personal communication, Netherlands: Leiden [2]
93		c.2724delT	p.Ser908fs	-	-	-	D.J.M. Peters, personal communication, Netherlands: Leiden [2]
94		c.2810dupC	p.Ser938fs	-	-	-	D.J.M. Peters, personal communication, Netherlands: Leiden [2]
95		c.4561-2A>G	p.? [3]	-	-	-	D.J.M. Peters, personal communication, Netherlands: Leiden [2]
96		c.4872dupC	p.Met1625fs	-	-	-	D.J.M. Peters, personal communication, Netherlands: Leiden [2]
97		c.5710C>T	p.Gln1904X	-	-	-	D.J.M. Peters, personal communication, Netherlands: Leiden [2]
98		c.4394+5G>T	p.? [3]	-	-	-	Gervasini, personal communication, Netherlands: Leiden [2]
99		c.4133G>C	p.Arg1378Cys	-	-	-	Gervasini, personal communication, Netherlands: Leiden [2]
100		c.317dupA	p.Pro107fs	-	-	-	Gervasini, personal communication, Netherlands: Leiden [2]
101		c.2356delC	p.Gln786fs	-	-	-	Gervasini, personal communication, Netherlands: Leiden [2]
102		c.3546delA	p.Glu1183fs	-	-	-	Gervasini, personal communication, Netherlands: Leiden [2]
103		c.3608_3609+5del	p.? [3]	-	-	-	Gervasini, personal communication, Netherlands: Leiden [2]
104		c.3751delC	p.Leu1251fs	-	-	-	Gervasini, personal communication, Netherlands: Leiden [2]
105		c.4400delT	p.Val1467fs	-	-	-	Gervasini, personal communication, Netherlands: Leiden [2]
106		c.4492C>T	p.Arg1498X	-	-	-	Gervasini, personal communication, Netherlands: Leiden [2]
107		c.5837dupC	p.Pro1947fs	-	-	-	Gervasini, personal communication, Netherlands: Leiden [2]

Table S2. *Cont.*

Index	Phenotype	cDNA Change	A.A. Variant	Origin	OMIM ID	dbSNP	Reference
108		c.5837delC	p.Pro1946fs	-	-	-	Gervasini, personal communication, Netherlands: Leiden [2]
109		c.5838_5857dup20	p.Pro1953fs	-	-	-	Gervasini, personal communication, Netherlands: Leiden [2]
110		c.3779+5G>C	p.? [3]	-	-	-	Kalkhoven 2004 [56]
111		c.4991G>A	p.Arg1664His	-	-	-	Kalkhoven 2004 [56]
112		c.4133+1G>A	p.? [3]	-	-	-	Kalkhoven 2004 [56]
113		c.4669C>T	p.Glu1557X	-	-	-	Kalkhoven 2004 [56]
114		c.4837delG	p.Val1613fs	-	-	-	Kalkhoven 2004 [56]
115		c.5837dupC	p.Pro1947fs	-	-	-	M.J. van Belzen [2]
116		c.3832G>A	p.Glu1278Lys	-	-	-	M.J. van Belzen, Netherlands: Leiden [2]
117		c.3832G>A	p.Glu1278Lys	-	-	-	M.J. van Belzen, Netherlands: Leiden [2]
118		c.(?_-204)_(*2664_?)del	p.? [3]	-	-	-	M.J. van Belzen, Netherlands: Leiden [2]
119		c.86-?_798+?del	p.? [3]	-	-	-	M.J. van Belzen, Netherlands: Leiden [2]
120		c.277dupA	p.Ser93fs	-	-	-	M.J. van Belzen, Netherlands: Leiden [2]
121		c.1069C>T	p.Gln357X	-	-	-	M.J. van Belzen, Netherlands: Leiden [2]
122		c.1237C>T	p.Arg413X	-	-	-	M.J. van Belzen, Netherlands: Leiden [2]
123		c.1270C>T	p.Arg424X	-	-	-	M.J. van Belzen, Netherlands: Leiden [2]
124		c.1318C>T	p.Arg440X	-	-	-	M.J. van Belzen, Netherlands: Leiden [2]
125		c.1655delC	p.Pro552fs	-	-	-	M.J. van Belzen, Netherlands: Leiden [2]
126		c.2842C>T	p.Gln948X	-	-	-	M.J. van Belzen, Netherlands: Leiden [2]
127		c.2842C>T	p.Gln948X	-	-	-	M.J. van Belzen, Netherlands: Leiden [2]
128		c.2879delC	p.Pro960fs	-	-	-	M.J. van Belzen, Netherlands: Leiden [2]
129		c.3061-?_3698+?del	p.? [3]	-	-	-	M.J. van Belzen, Netherlands: Leiden [2]
130		c.3375T>G	p.Tyr1125X	-	-	-	M.J. van Belzen, Netherlands: Leiden [2]
131		c.3547G>T	p.Glu1183X	-	-	-	M.J. van Belzen, Netherlands: Leiden [2]
132		c.3610-2A>G	p.? [3]	-	-	-	M.J. van Belzen, Netherlands: Leiden [2]
133		c.3610-1G>A	p.? [3]	-	-	-	M.J. van Belzen, Netherlands: Leiden [2]
134		c.3779+2T>C	p.? [3]	-	-	-	M.J. van Belzen, Netherlands: Leiden [2]

Table S2. *Cont.*

Index	Phenotype	cDNA Change	A.A. Variant	Origin	OMIM ID	dbSNP	Reference
135		c.3817_3821dupGATAC	p.Leu1275fs	-	-	-	M.J. van Belzen, Netherlands: Leiden [2]
136		c.3862_3871del	p.Arg1288fs	-	-	-	M.J. van Belzen, Netherlands: Leiden [2]
137		c.3914+1G>T	p.? [3]	-	-	-	M.J. van Belzen, Netherlands: Leiden [2]
138		c.3983-?_(*2664_?)del	p.? [3]	-	-	-	M.J. van Belzen, Netherlands: Leiden [2]
139		c.4268dupC	p.Pro1424fs	-	-	-	M.J. van Belzen, Netherlands: Leiden [2]
140		c.4396_4406del	p.Tyr1466fs	-	-	-	M.J. van Belzen, Netherlands: Leiden [2]
141		c.4567_4568delTT	p.Phe1523fs	-	-	-	M.J. van Belzen, Netherlands: Leiden [2]
142		c.5641_5642delAG	p.Leu1882fs	-	-	-	M.J. van Belzen, Netherlands: Leiden [2]
143		c.5821C>T	p.Gln1941X	-	-	-	M.J. van Belzen, Netherlands: Leiden [2]
144		c.6010C>T	p.Arg2004X	-	-	-	M.J. van Belzen, Netherlands: Leiden [2]
145		c.6213delG	p.Arg2072fs	-	-	-	M.J. van Belzen, Netherlands: Leiden [2]
146		c.6436C>T	p.Gln2146X	-	-	-	M.J. van Belzen, Netherlands: Leiden [2]
147		c.5212_5213ins14	p.His1738fs	-	-	-	Murata 2001 [52]
148		c.5222_5223delAG	p.Lys1741fs	-	-	-	Murata 2001 [52]
149		c.86-?_798+?del	p.? [3]	-	-	-	Netherlands: Leiden
150		c.4340C>T	p.Thr1447Ile	-	-	-	Roelfsema 2005 [56]
151		c.4348T>C	p.Tyr1450His	-	-	-	Roelfsema 2005 [56]
152		c.4409A>G	p.His1470Arg	-	-	-	Roelfsema 2005 [56]
153		c.235delG	p.Gly79fs	-	-	-	Roelfsema 2005 [56]
154		c.304C>T	p.Gln102X	-	-	-	Roelfsema 2005 [8]
155		c.904_905delAG	p.Ser302fs	-	-	-	Roelfsema 2005 [56]
156		c.1237C>T	p.Arg413X	-	-	-	Roelfsema 2005 [56]
157		c.1481dupA	p.Asn494fs	-	-	-	Roelfsema 2005 [56]
158		c.3396_3400delCATGG	p.Pro1133fs	-	-	-	Roelfsema 2005 [56]
159		c.3432_3433delAG	p.Gly1145fs	-	-	-	Roelfsema 2005 [56]
160		c.3824dupT	p.Leu1275fs	-	-	-	Roelfsema 2005 [56]
161		c.3915-1G>A	p.? [3]	-	-	-	Roelfsema 2005 [56]

Table S2. *Cont.*

Index	Phenotype	cDNA Change	A.A. Variant	Origin	OMIM ID	dbSNP	Reference
162		c.4398dupT	p.Val1467fs	-	-	-	Roelfsema 2005 [56]
163		c.4879A>T	p.Lys1627X	-	-	-	Roelfsema 2005 [56]
164		c.6010C>T	p.Arg2004X	-	-	-	Roelfsema 2005 [56]
165		c.6133C>T	p.Gln2045X	-	-	-	Roelfsema 2005 [56]
166		c.6283C>T	p.Gln2095X	-	-	-	Roelfsema 2005 [56]
167		c.1388_1395delGGCAACAG	p.Gly463fs	-	-	-	Roelfsema 2005 [56], Netherlands: Leiden [2]
168		c.1735dupA	p.Thr579fs	-	-	-	Roelfsema 2005 [56], Netherlands: Leiden [2]
169		c.3833A>G	p.Glu1278Gly	-	-	-	Udaka 2005 [57]
170		c.4238A>C	p.His1413Pro	-	-	-	Udaka 2005 [57]
171		c.1108C>T	p.Arg370X	-	-	-	Udaka 2005 [57]
172		c.1270C>T	p.Arg424X	-	-	-	Udaka 2005 [57]
173		c.1733delC	p.Pro578fs	-	-	-	Udaka 2005 [57]
174		c.2749dupA	p.Thr917fs	-	-	-	Udaka 2005 [57]
175		c.3545dupC	p.Glu1183fs	-	-	-	Udaka 2005 [57]
176		c.3698+1G>A	p.? [3]	-	-	-	Udaka 2005 [57]
177		c.3836+1G>A	p.? [3]	-	-	-	Udaka 2005 [57]
178		c.4492C>T	p.Arg1498X	-	-	-	Udaka 2005 [57]
179		c.4611delG	p.Tyr1539fs	-	-	-	Udaka 2005 [57]
180		c.5793dupC	p.Thr1932fs	-	-	-	Udaka 2005 [57]

[1] Variant of Amino Acid. Protein change predicted from RNA; [2] Do not have reference. But have LOVD submitter ID; [3] Unknown. Protein change not predicted from RNA; OMIM ID: OMIM (Online Mendelian Inheritance in Man) allelic variant ID in CREBBP; dbSNP: dbSNP (Single Nucleotide Polymorphism Database) variant ID. The rs number is an accession number used by researchers and databases to refer to specific SNPs. It stands for Reference SNP cluster ID; and Hyphens (-) in table mean not-available or not-found.
